# Hematopoietic Stem Cell-derived Adipocytes Promote Tumor Growth and Cancer Cell Migration

**DOI:** 10.16966/2381-3318.130

**Published:** 2017-03-08

**Authors:** Y Xiong, DL Russell, LT McDonald, LA Cowart, AC LaRue

**Affiliations:** 1 Research Services, Ralph H Johnson Veterans Affairs Medical Center, Charleston, South Carolina, USA; 2 Department of Pathology and Laboratory Medicine, Medical University of South Carolina, Charleston, South Carolina, USA; 3 The Hollings Cancer Center, Medical University of South Carolina, Charleston, South Carolina, USA; 4 Department of Biochemistry and Molecular Biology, Medical University of South Carolina, Charleston, South Carolina, USA

**Keywords:** Hematopoietic stem cell, Adipocyte, Melanoma, Breast cancer, Tumor microenvironment

## Abstract

Adipocytes, apart from their critical role as the energy storage depots, contribute to the composition of the tumor microenvironment. Our previous studies based on a single hematopoietic stem cell (HSC) transplantation model, have revealed a novel source of adipocytes from HSCs *via* monocyte/macrophage progenitors. Herein, we extend these studies to examine the role of HSC-derived adipocytes (HSC-Ad) in tumor progression. When cultured under adipogenic conditions, bone marrow-derived monocytic progenitors differentiated into adipocytes that accumulated oil droplets containing triglyceride. The adipokine array and ELISAs confirmed secretion of multiple adipokines by HSC-Ad. These adipocytes underwent further development *in vivo* when injected subcutaneously into C57Bl/6 mice. When co-injected with melanoma B16F1 cells or breast cancer E0771 cells into syngeneic C57Bl/6 mice, HSC-Ad not only accelerated both melanoma and breast tumor growth, but also enhanced vascularization in both tumors. Conditioned media from HSC-Ad supported B16F1 and E0771 cell proliferation and enhanced cell migration *in vitro*. Among the HSC-Ad secreted adipokines, insulin-like growth factor 1 (IGF-1) played an important role in E0771 cell proliferation. Hepatocyte growth factor (HGF) was indispensable for B16F1 cell migration, whereas HGF and platelet-derived growth factor BB (PDGF-BB) collectively contributed to E0771 cell migration. Expression levels of receptors for IGF-1, HGF, and PDGF-BB correlated with their differential roles in B16F1 and E0771 cell proliferation and migration. Our data suggest that HSC-Ad differentially regulate tumor behavior through distinct mechanisms.

## Introduction

Tumor cells are capable of growth only if the complex tissue environment provides them with a permissive and supportive milieu. Thus, tumor proliferation, invasiveness, angiogenesis, and metastatic potential are dependent on a complicated bidirectional crosstalk between tumor cells and cells of the tumor microenvironment. Cells that compose the tumor stroma include fibroblasts and adipocytes, recruited immune cells, and endothelial cells in newly formed blood vessels [1]. Adipocytes are present in multiple tissues, some of which have an adipocyte-rich environment, such as the mammary gland and bone marrow. To date, most of the studies focused on cancer cell-stromal cell communications have emphasized the contributions of fibroblasts, endothelial cells, and inflammatory cells, whereas the role of adipocytes in tumor progression requires more in-depth exploration.

Emerging evidence reveals that, apart from its critical role as the largest repository of energy, adipocytes are endocrine cells, producing hormones, growth factors, chemokines, and adipokines that influence tumor growth, metastasis, and chemoresistance in a variety of cancer types [2]. For example, the contributions of adipocytes to breast cancer growth and metastasis have been extensively examined [3–5]. Recently, adipocytes have been shown to provide lipids, in the form of free fatty acids, to cancer cells in immediate proximity for their energy production [6,7]. During their interaction with cancer cells, adipocytes acquire phenotypic changes (e.g. de-lipidation, de-differentiation) and functional alterations (e.g. increased production of interleukin-6), and are thus referred to as cancer-associated adipocytes [8,9]. It has been shown that cancer-associated adipocytes may provide elevated levels of pro-inflammatory cytokines [4], proteases [10,11], and extracellular matrix constituents [12,13] to cancer cells, thus, accelerating tumor progression.

The generation of new adipocytes from progenitor cells has been of great interest in terms of understanding adipose tissue development and turnover under normal or pathological conditions, such as obesity and cancer. Adipocytes are generally believed to arise from resident adipose tissue pre-adipocytes and mesenchymal stem cells (MSCs) [14,15]. Several groups, including our laboratory, have discovered a novel adipocyte population originating from bone marrow-derived circulating progenitor cells [16–18]. Bone marrow provides a rich source for both MSCs and hematopoietic stem cells (HSCs). In our laboratory, transplantation of a clonal population of cells derived from a single EGFP-positive HSC demonstrated an HSC origin of adipocytes that differentiated *via* monocyte/macrophage progenitors [18]. This was supported by the work from the Klemm group, who used a non-transplant transgenic mouse model in which LacZ expression is restricted to the myeloid lineage that confirmed the *de novo* generation of a subset of adipocytes from bone marrow hematopoietic/myeloid progenitor cells [19].

To better understand the contribution of HSC-derived adipocyte (HSC-Ad) to tumor progression, we have enriched for the bone marrow-derived monocytic progenitors and differentiated them into adipocytes. We have further analyzed their influences on tumor behavior, particularly, tumor growth and cancer cell motility. In the present study, we demonstrate that HSC-Ad secrete growth factors and adipokines that differentially regulate melanoma and breast tumor growth and cancer cell migration. We have shown that these effects are dependent on the involvement of distinct signaling pathways in the tumor models.

## Materials and Methods

### Mice

C57Bl/6-CD45.1 breeders were purchased from Jackson Laboratories (Bar Harbor, ME). Transgenic EGFP breeding pairs (C57Bl/6-CD45.2 background) were provided by Dr. Masaru Okabe [20] (Osaka University, Japan). Mice were bred and maintained at the Animal Research Facility of the Veterans Affairs Medical Center (Charleston, South Carolina). Research was conducted in accordance with guidelines set by the PHS Policy on Human Care and the Use of Laboratory Animals and the Institutional Animal Care and Use Committee, Department of Veterans Affairs Medical Center.

### Cell lines

The murine melanoma cell line B16F1 was purchased from American Type Culture Collection (ATCC, Manassas, VA) and the murine breast cancer cell line E0771 was a gift from Dr. Dennis Watson (MUSC, Charleston, SC). For this study both cell lines were maintained in low passage (<10) at 37°C in a humidified atmosphere of 5% CO_2_. B16F1 cells were cultured in Dulbecco’s Modified Eagle medium (DMEM) containing 10% fetal bovine serum (FBS). E0771 cells were cultured in DMEM containing 20% FBS.

### Reagents

Cell culture media were purchased from Life Technologies (Grand Island, NY). FBS was from Atlanta Biologicals (Flowery Branch, Georgia). Recombinant proteins and neutralizing antibodies were purchased from R&D systems (Minneapolis, Minnesota). Phycoerythrin (PE)-conjugated anti-F4/80 (T45-2342) and allophycocyanin (APC)-conjugated anti-CD11b (M1/70) antibodies were from BD Biosciences (San Jose, California). PE-conjugated anti-c-Met (eBioclone 7) antibody was from eBioscience (San Diego, California). CD31 antibody was from Abcam (Cambridge, Massachusetts). Antibodies used in western blot were purchased from Cell Signaling Technology (Danvers, Massachusetts). Inhibitors were purchased from Selleckchem (Houston, Texas).

### Adipogenesis of monocytic precursors *in vitro*

The primitive mononuclear precursor cells were generated and expanded as described by Stanley and colleagues [21]. Briefly, mouse bone marrow cells were extracted and cultured in minimum essential medium α (αMEM) containing 20% FBS, 15 ng/mL interleukin-3 (IL-3), and 0.6 ng/mL macrophage colony stimulating factor (M-CSF) for a period of 3 days. The non-adherent cells were treated with 0.02% Pronase (EMD Millipore) and then cultured in αMEM containing 20% FBS and 10 ng/mL of M-CSF for a period of 2 days. The resulting adherent cells were either supplemented with 100 ng/mL of M-CSF for 3 days to differentiate into macrophages or subjected to adipogenic conditions described below.

To differentiate the adherent cells into adipocytes, complete growth medium was replaced with adipogenesis initiation medium consisting of αMEM, 10% FBS, 10% mouse serum (MS), 100 ng/mL of M-CSF, 0.5 mM isobutylmethylxanthine, 1 μM dexamethasone, and 10 μg/mL of insulin. After incubation for 2 days, the medium was replaced with adipogenesis progression medium containing αMEM, 10% FBS, 10% MS, 100 ng/mL M-CSF, and 10 μg/mL of insulin. Two days later, the medium was replaced with adipogenesis maintenance medium containing αMEM, 10% FBS, 10% MS, and 100 ng/mL of M-CSF and incubation continued for at least 5 days. For adipocyte conditioned medium (Ad-CM), cells were rinsed twice with phosphate buffered saline (PBS), and αMEM was subsequently added. After 24 h, the medium was collected, centrifuged at 2,000 rpm for 5 min, and the supernatant was used to quantify the secreted adipokines and determine the effect on tumor cell proliferation and migration.

### Extraction of mature adipocytes

Subcutaneous adipose tissue was excised and minced in DMEM/F12 medium containing 1 mg/mL of type I collagenase (Sigma, St. Louis, Missouri) and 0.1% bovine serum albumin (BSA), and incubated at 37°C on a rotary shaker at 100 rpm for 1 h. Undigested tissue was removed after filtration through a 150-μm mesh filter, followed by the separation of mature adipocytes from stromal vascular cells through centrifugation at 200 g for 5 min. Mature adipocytes (top layer) were collected and subjected to ceiling culture [22].

### Determination of lipid content and adipokine secretion

Triglyceride was extracted from cells with 5% NP-40 and quantified using a triglyceride determination kit (Abcam) according to the manufacturer’s protocol. Secreted adipokines in Ad-CM were quantified using individual Quantikine ELISA kits (R&D Systems). Data was analyzed with a SPECTRAmax M2 microplate reader running Soft Max Pro software (Molecular Devices, Sunnyvale, CA).

### Mouse orthotopic tumor experiments

B16F1 (2 × 10^5^) cells were washed with serum-free medium and re-suspended with or without HSC-Ad (8 × 10^5^) in 200 μL of Matrigel (Corning, Tewksbury, Massachusetts) and injected subcutaneously into C57Bl/6-CD45.1 mice. E0771 (2 × 10^5^) cells were washed with serum-free medium and re-suspended with or without HSC-Ad (8 × 10^5^) in 50 μL of Matrigel and injected into 4^th^ mammary glands of C57Bl/6-CD45.1 female mice. Tumor area was measured during tumor growth (Length × Width), and tumor volume was measured at the end point when mice were euthanized (0.5 × Length × Width × Height). In orthotopic models, different tumor-to-stroma ratios were examined: 1:1 versus 1:4. We observed that B16F1/E0771: HSC-Ad ratio 1:1 did not present a significant effect in accelerating tumor growth in the timeframe examined, thus a tumor-to-stroma ratio of 1:4 was used for *in vivo* experiments.

### Immunohistochemistry and vessel quantification

Tumor sections (5 μM) were deparaffinized in xylenes and dehydrated in alcohol according to standard procedures. Sections were stained with antibodies to CD31 using a rabbit IgG kit (Vector laboratories) and counterstained with hematoxylin. Sections from three tumors per group were imaged on a Nikon Eclipse 90i microscope. The number and lumen area of blood vessels were quantified and averaged from 3–4 high power fields (HPF, 200X) per section.

### Cell proliferation and migration assays

For cell proliferation assays, tumor cells were seeded in triplicate at a density of 3 × 10^3^ cells per well in 96-well plates and quantified over 3 days using a CyQUANT NF Cell Proliferation Assay Kit (Life Technologies) according to the manufacturer’s protocol. Migration assays were performed using 8.0 μm pore size Costar Transwell inserts (Corning) coated with human fibronectin. B16F1 (3 × 10^4^) or E0771 (5 × 10^4^) cells were re-suspended in 100 μL of serum-free medium and seeded on fibronectin-coated insert membranes. Experimental medium was placed in the wells beneath the inserts. After 4 h incubation in 5% CO_2_ at 37°C, non-migrated cells were removed with a cotton swab and cells that migrated to the other side of the membrane were fixed and stained with Diff-Quick kit (Siemens Healthcare Diagnostics, Malvern, PA). Cell numbers were counted and averaged from 10 HPFs per insert.

### FACS analysis

Bone marrow-derived monocytic cells were stained with APC-conjugated anti-CD11b and PE-conjugated anti-F4/80 antibodies. Tumor cells were stained with PE-conjugated anti-c-Met antibody. Propidium iodide (PI)-negative live cells were analyzed using a FACSCalibur (Becton Dickinson).

### Western blot

Cells were harvested in lysis buffer consisting of 50 mM Tris, pH 7.4, 150 mM NaCl, 1% NP-40, and 5 mM EDTA. Following 30 min of incubation in lysis buffer at 4°C, lysates were cleared by centrifugation at 13,000 rpm for 10 min at 4°C. Supernatants were collected and protein concentrations determined using BCA protein assay kit (Thermo Fisher Scientific). Total proteins (40 μg) were loaded and run on SDS-PAGE gel, transferred to PVDF membrane, blocked in 5% milk and blotted with appropriate antibodies and HRP-conjugated secondary antibodies before incubation with ECL (Bio-Rad) and film exposure.

### Statistical analysis

Statistical analysis was performed using Excel. For the *in vitro* experiments, all data was presented as the mean ± S.D. where appropriate. The statistical significance of differences between means (at least 3 independent experiments) was evaluated using two-tailed Student’s *t* test. The values of ^*^p≤0.05, ^**^p*<*0.01, and ^***^p*<*0.001 were deemed as significant whereas “n.s.” denoted not significant.

## Results

### Monocytic precursors give rise to adipocytes

**Figure 1:**
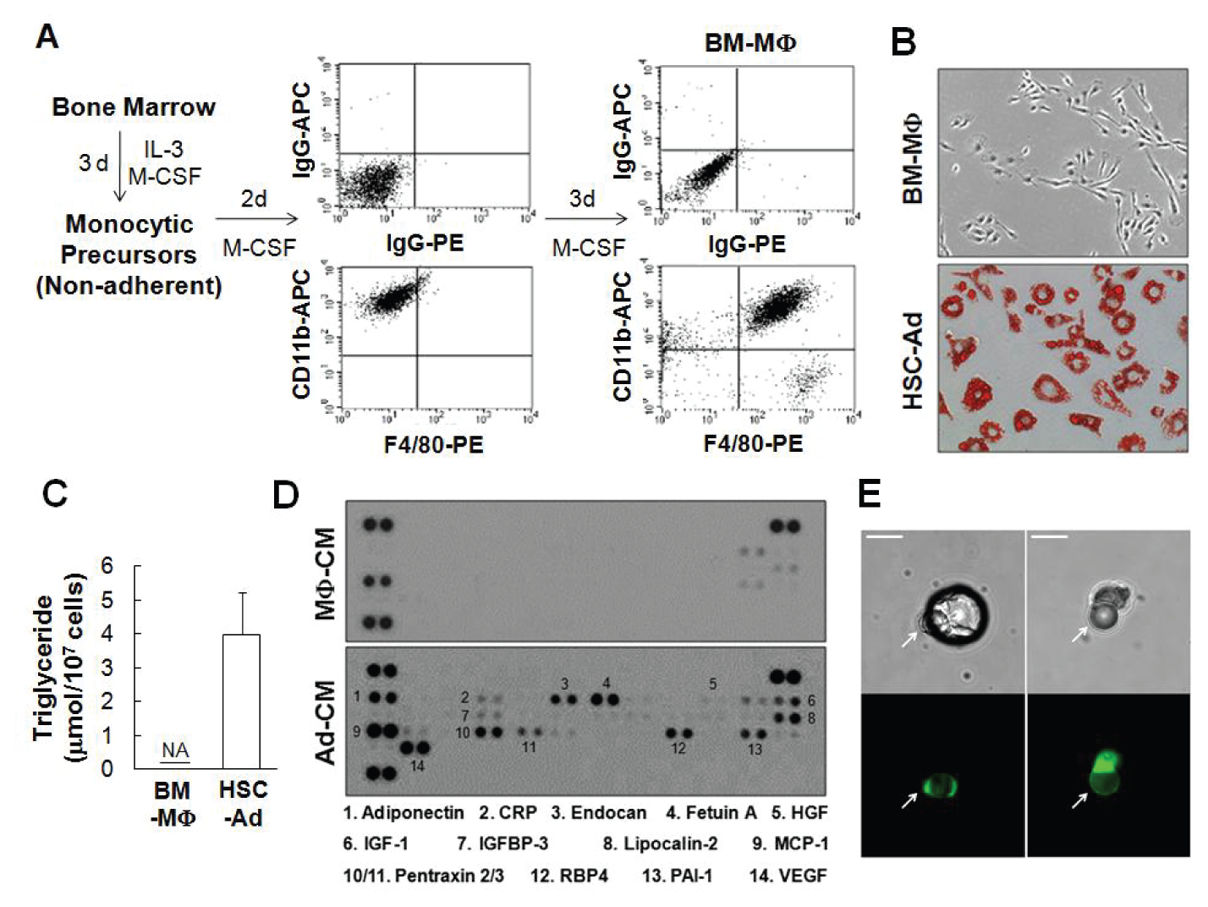
Monocytic precursors give rise to adipocytes in vitro and in vivo-(A) Enrichment of monocytic precursor from bone marrow in the presence of interleukin-3 (IL-3) and macrophage colony-stimulating factor (M-CSF). FACS analysis of CD11b and F4/80 staining demonstrated the differentiation of macrophages from monocytic precursors. (B) Bone marrow-derived macrophages (BM-MΦ), and adipocytes differentiated from hematopoietic stem cell (HSC) via monocytic precursors (HSC-Ad), were stained with Oil Red O (ORO) and visualized with phase contrast imaging. (C) Triglyceride contents were extracted from BM-MΦ and HSC-Ad, and quantified. NA, not detected. (D) BM-MΦ or HSC-Ad were starved overnight and the resulting medium was used as conditioned medium, denoted as MΦ-CM or Ad-CM. MΦ-CM and Ad-CM were subjected to an Adipokine Array (R&D Systems), and the profiles of secreted adipokines were compared. (E) EGFP^+^ HSC-Ad were embedded in Matrigel and injected subcutaneously into C57Bl/6 mice. After 3 weeks, mature adipocytes were extracted, cultured and imaged. Arrows indicated the mature EGFP^+^ adipocytes from HSC origin. Scale bars=25 μm.

Using transplant [18] and non-transplant [19] mouse models, we and others have shown that hematopoietic stem cells (HSCs) are able to differentiate into adipocytes *via* progenitors for monocytes/macrophages. To further extend this initial observation, primitive monocytic progenitor cells from bone marrow were generated and expanded as established by Stanley et al. [21] and described in Materials and Methods (Figure 1A). As a proof of concept, after 2 days of culture in 10 ng/mL of macrophage colony-stimulating factor (M-CSF), non-adherent cells became adherent and were CD11b^high^F4/80^-/dim^ (Figure 1A-middle panels). In response to an additional 3 days of culture in 100 ng/mL of M-CSF, the adherent cells exhibited a high level of F4/80 expression (Figure 1A-right panels) in addition to an elongated morphology (Figure 1B-top panel), both indicative of cellular differentiation into mature bone marrow-derived macrophages (BM-MΦ). However, when subjected to adipogenic conditions, these HSC-derived cells stained positive for Oil Red O (ORO) (Figure 1B-bottom panel) and accumulated multiple lipid droplets containing triglyceride (Figure 1C). BM-MΦ cells were ORO-negative (Figure 1B-top panel) and did not store any triglyceride (Figure 1C). HSC-derived adipocyte (HSC-Ad) also secreted adipokines, including adiponectin, insulin-like growth factor-1 (IGF-1), monocyte chemotactic protein-1 (MCP-1), and vascular endothelial growth factor (VEGF) (Figure 1D, Supplementary Figure S1). Although hepatocyte growth factor (HGF) was barely detectable by an Adipokine Array (Figure 1D), yet its presence was confirmed with HGF ELISA (Supplementary Figure S1). These results demonstrated that monocytic progenitor cells are able to differentiate into adipocytes *in vitro*.

To determine whether HSC-Ad could undergo further maturation and form one large central droplet of fat in their cytoplasm, monocytic progenitor cells were generated from bone marrow of EGFP mice and were differentiated into adipocytes *in vitro*. These EGFP-positive adipocytes were embedded in Matrigel and the mixture was injected subcutaneously into C57Bl/6 mice. Mature adipocytes were extracted from adipose tissue at the site of implantation 3 weeks post-implantation and subjected to ceiling culture [22]. After 2 days, isolated adipocytes loosely adhered to the ceiling surface and the culture was imaged thereafter. Several mature adipocytes with unilocular morphology (Figure 1E-upper panels) were positive for EGFP fluorescence (Figure 1E-bottom panels), suggesting that, under a favorable microenvironment, HSC-Ad can fully develop and integrate into adipose tissue.

### HSC-Adipocytes promote melanoma and breast tumor growth

The effect of HSC-Ad on tumor growth *in vivo* was next examined. Two types of murine tumor cell lines were used in this study, the melanoma cell line B16F1 and the breast cancer cell line E0771. Tumor cells were injected orthotopically (subcutaneous-B16F1; mammary fat pad-E0771) into syngeneic C57Bl/6 mice, alone or with HSC-Ad at a ratio of 1:4. Tumors were allowed to progress to day 14 for B16F1 and day 32 for E0771, due to IACUC approved humane endpoint where tumor size may not exceed 2 cm in diameter.

**Figure 2:**
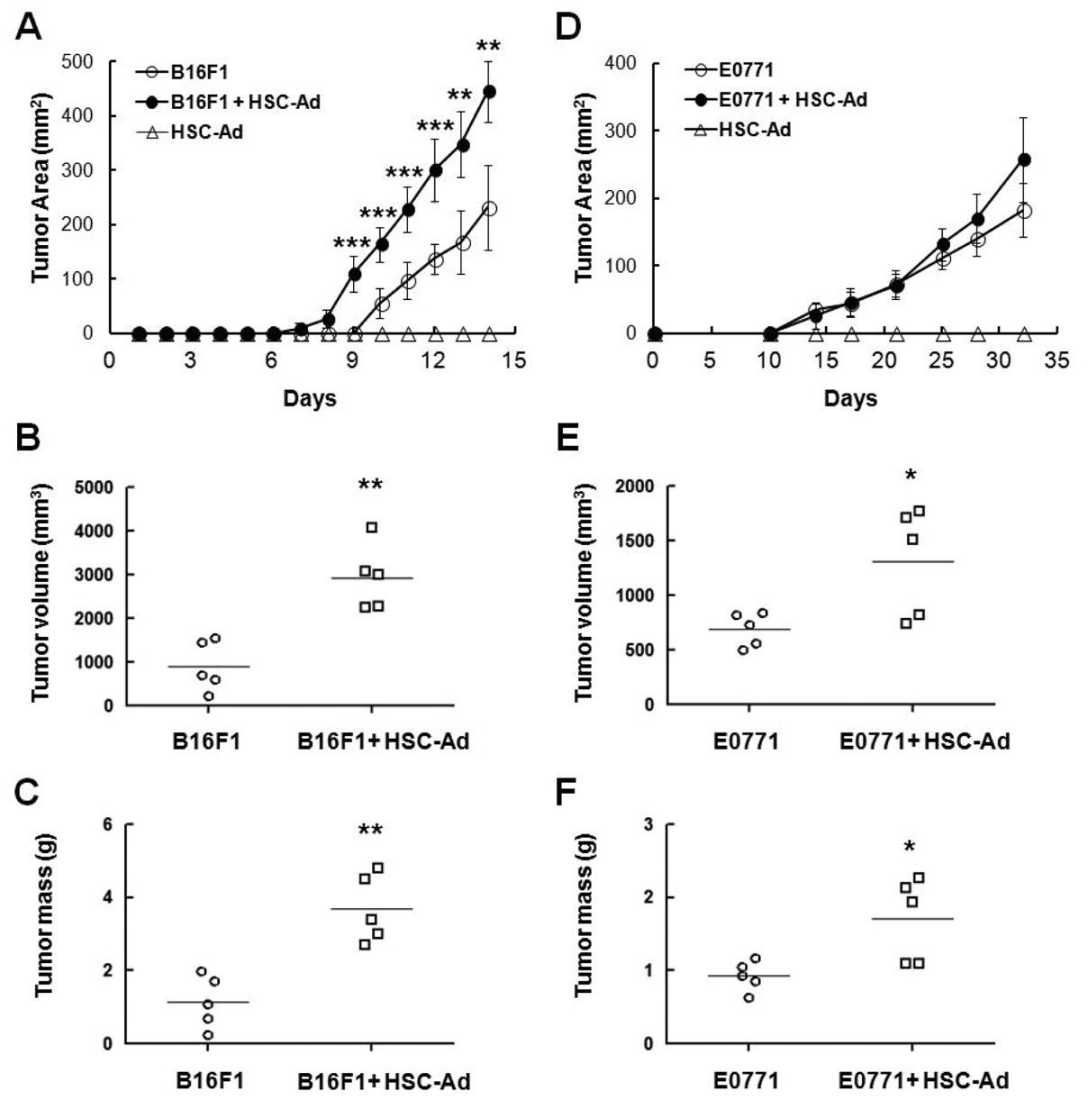
HSC-derived adipocytes accelerate melanoma (B16F1) and breast tumor (E0771) growth *in vivo***-**Tumor cells alone ( 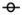), HSC-Ad alone ( 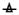 ), or both ( 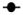) were injected orthotopically into C57Bl/6 mice. Tumor area was measured on a regular basis (A, D). At the end point when mice were euthanized, tumor volume (B, E) and mass (C, F) were measured. (A-C) B16F1 tumor growth. (D-F) E0771 tumor growth. ^*^p≤0.05, ^**^p<0.01, ^***^p<0.001.

In the melanoma model, tumor size was measured on a daily basis and mice euthanized on day 14. When injected alone, B16F1 tumors were measurable on day 10 (Figure 2A). With HSC-Ad co-injection, B16F1 tumors were measurable on day 8 (Figure 2A). Moreover, co-injection of HSC-Ad resulted in significantly larger (2.7-fold, p<0.01) (Figure 2B) and heavier (2.7-fold, p<0.01) (Figure 2C) melanoma tumors at the endpoint. HSC-Ad not only promoted B16F1 tumor initiation, but also accelerated tumor growth rate as B16F1 tumor growth rate was significantly elevated (p<0.01), from 43.85 ± 13.17 mm^2^/day when injected alone, to 69.53 ± 8.44 mm^2^/day when co-injected with HSC-Ad (Figure 2A).

With regard to breast tumor growth, tumor size was measured twice a week and mice euthanized on day 32 based on approved humane endpoint. Injection of E0771 alone formed measurable tumors at day 10, as did E0771-HSC-Ad co-injection (Figure 2D). Unlike B16F1 tumors, E0771 tumor initiation was not affected by co-injection of HSC-Ad, which nevertheless showed a trend of accelerated E0771 tumor growth that led to significantly larger (1.9-fold, p<0.05) (Figure 2E) and heavier (1.8-fold, p<0.05) (Figure 2F) breast tumors at the endpoint. Injections of HSC-Ad alone did not generate tumor in either model (Figures 2A and 2D).

### HSC-Adipocytes enhance tumor angiogenesis *in vivo*

**Figure 3:**
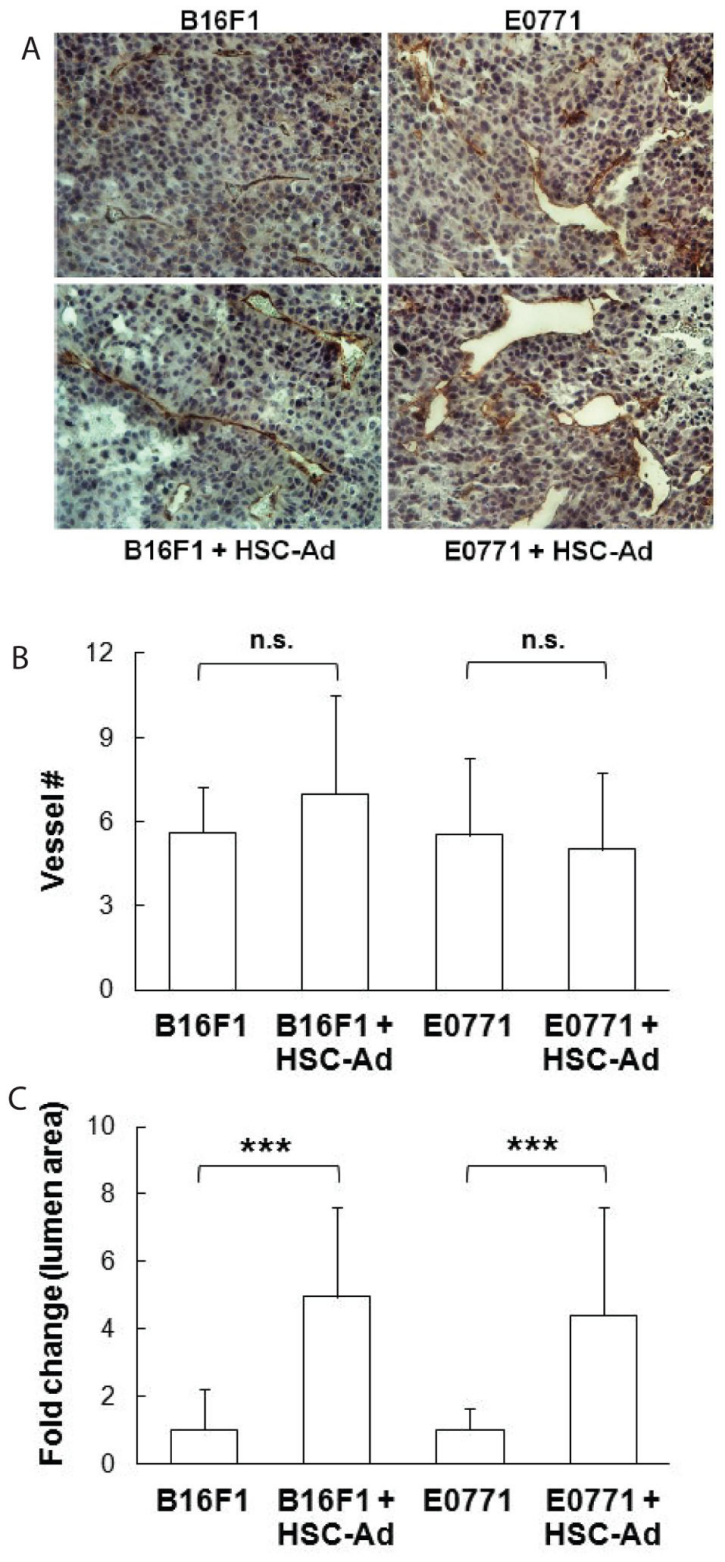
HSC-derived adipocytes enhance B16F1 and E0771 tumor vascularization *in vivo***-**Tumor cells were orthotopically injected into C57Bl/6 mice, either alone or combined with HSC-Ad. (A) Tumor sections (5 μm) were stained with CD31 antibody to indicate blood vessels, and were counterstained with hemotoxylin. (B) Quantification of the number (#) of blood vessels. (C) Lumen area of blood vessels was measured and presented as fold change of co-injection *vs* injection of tumor cells only. Data was averaged from 3–4 high power fields (HPF, 200X) per section from 3 tumors for each group (n=3). ^***^p<0.001. n.s., not significant.

An important pro-tumorigenic process is angiogenesis, which acts to reinforce oxygen and nutrient supply to the tumor. We observed that HSC-Ad secreted the pro-angiogenic factor VEGF (Figure 1D), which led us to examine the effect of HSC-Ad on vascularization in melanoma and breast tumors. Paraffin sections (5 μm) of tumors were stained using antibodies to CD31, an endothelial cell marker (Figure 3A). Blood vessel number and lumen area were measured. Quantitative analysis showed that blood vessel numbers remained unchanged regardless of co-injection of HSC-Ad in both tumor models (Figure 3B). However, compared with injection of tumor cells alone, co-injection with HSC-Ad resulted in formation of blood vessels with significantly larger lumen area (~5-fold increase for melanoma, p<0.001; ~4.4-fold increase for breast tumor, p<0.001) (Figure 3C) in both tumor models. HSC-Ad seemed to have more influence on enlarging vessel volume in melanoma tumors, which agrees with B16F1 tumor growth being more responsive to HSC-Ad co-injection than E0771 tumor growth. These results suggest that HSC-Ad promote tumor growth, at least in part through enhancing tumor vascularization as reflected by enlarged blood vessel lumen area.

### HSC-Adipocytes support cancer cell proliferation through activation of ERK and AKT pathways *in vitro*

**Figure 4:**
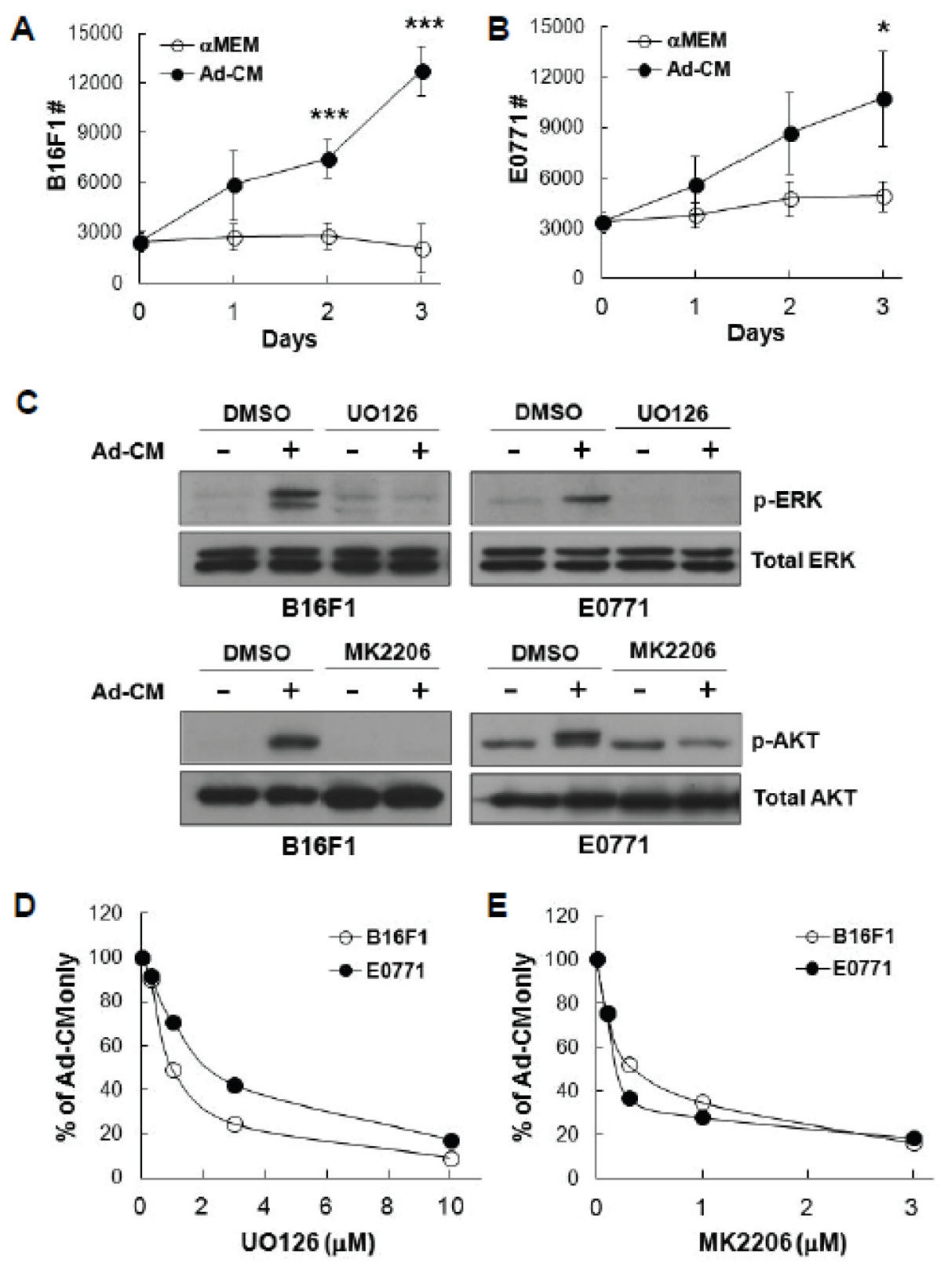
HSC-derived adipocytes support B16F1 and E0771 cell proliferation through activated ERK and AKT pathways *in vitro***-**(A) B16F1 and (B) E0771 cells were cultured in either serum-free αMEM ( 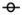)or Ad-CM ( 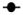) for 3 days and cell numbers (#) were quantified daily and averaged from multiple experiments. ^*^p<0.05, ^***^p<0.001. (C) Tumor cells were pretreated with DMSO or the indicated inhibitors (UO126, 10 μM; MK2206, 1 μM) overnight, followed by the addition of Ad-CM at 37°C for 1h. Cell lysates were subject to SDS-PAGE and blotted with the indicated antibodies. (D) B16F1 ( 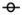) and E0771 ( 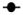) cells were cultured for 3 days in either Ad-CM or Ad-CM supplemented with different concentrations of UO126 or MK2206. Cell numbers were quantified on day 3. Data was averaged from two independent experiments and presented as percentage of Ad-CM.

In addition to the pro-angiogenic function of HSC-Ad on tumor growth *in vivo*, we next examined their effect on tumor cell proliferation *in vitro*. HSC-Ad were starved overnight and the resulting medium was used as conditioned medium (Ad-CM). B16F1 or E0771 cells were cultured in Ad-CM for up to 3 days and cell numbers were quantified daily. Both B16F1 (Figure 4A) and E0771 (Figure 4B) cells increased in number in response to Ad-CM, compared with cells maintained in serum-free αMEM. It is notable that B16F1 cells showed a higher proliferative rate than E0771 cells in the presence of Ad-CM (doubling time 30.24 ± 3.96 h *vs* 41.75 ± 1.71 h, p<0.05). This is in accordance with our finding that co-injection of HSC-Ad was more effective in accelerating B16F1 tumor growth than for E0771 tumor growth.

To address which downstream signals were regulating Ad-CM-supported tumor cell proliferation, we chose to examine the ERK and AKT pathways, as they are well-known regulators for cell survival and proliferation in general, as well as in melanoma and breast cancer progression [23–25]. We observed that addition of Ad-CM to B16F1 and E0771 cells resulted in ERK phosphorylation, which was completely abolished with MEK/ERK inhibitor UO126 (Figure 4C-upper panels). Likewise, Ad-CM also induced phosphorylation of AKT, which was abrogated by AKT inhibitor MK2206 (Figure 4C-lower panels). When tumor cells were cultured in Ad-CM in the presence of UO126 or MK2206, Ad-CM-supported cell proliferation was inhibited at day 3 in a dose-dependent manner in both tumor models (Figure 4D and 4E). These findings indicate that activation of ERK and AKT pathways are involved in B16F1 and E0771 cell proliferation in response to Ad-CM stimulation.

**Figure 5:**
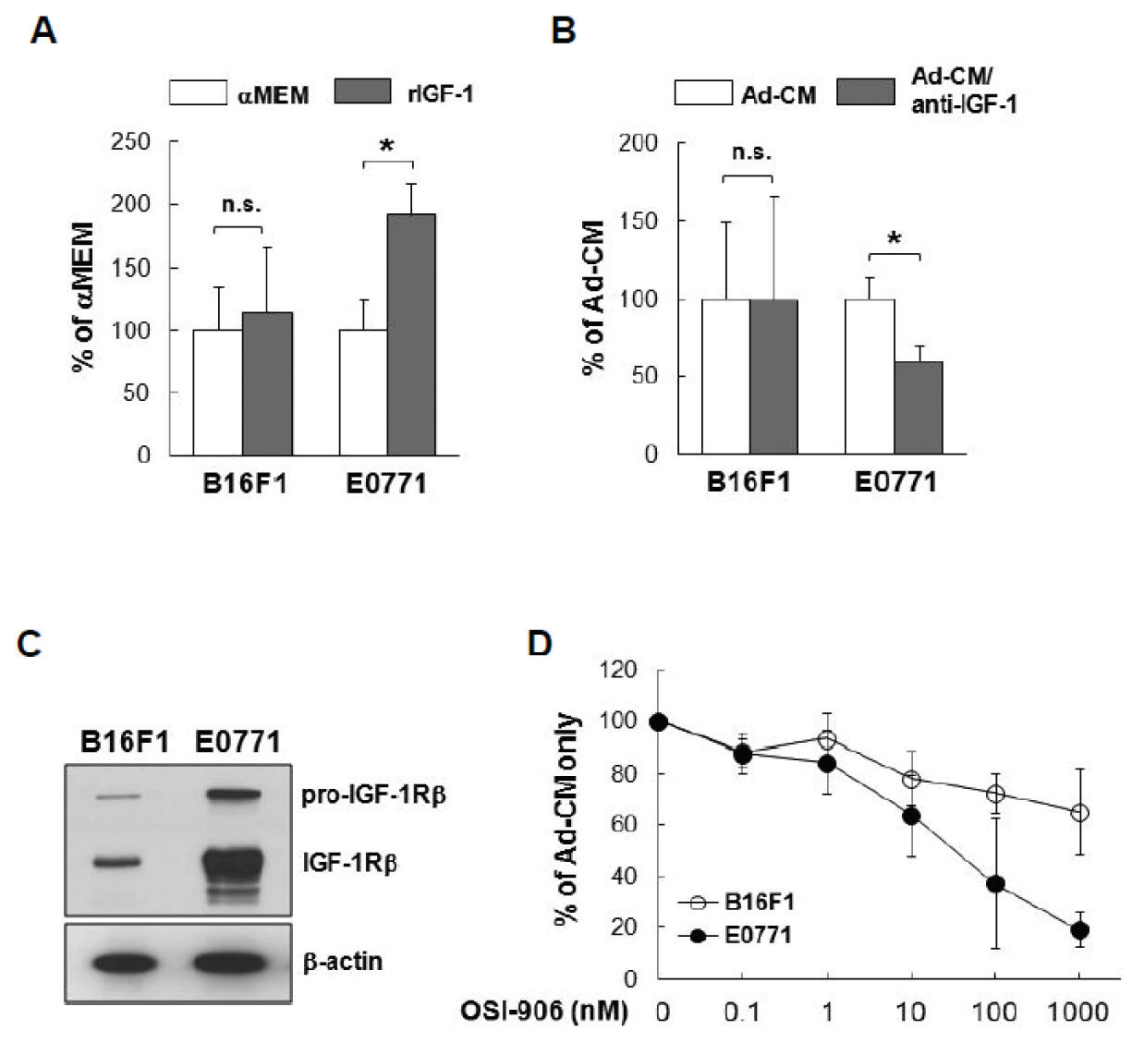
Insulin-like growth factor 1 (IGF-1)/IGF-1 receptor (IGF-1R) signal axis is responsible for HSC-Ad-stimulated E0771 cell proliferation *in vitro-*(A) Tumor cells were cultured for 3 days in either serum-free MEM or αMEM supplemented with 100 ng/mL of recombinant insulin growth factor-1 (rIGF-1). Cell numbers were quantified on day 3 and averaged from multiple experiments. Data was presented as percentage of αMEM. (B) Tumor cells were cultured for 3 days in either Ad-CM or Ad-CM supplemented with 1 μg/mL of IGF-1 neutralizing antibodies. Cell numbers were quantified on day 3 and averaged from multiple experiments. Data was presented as percentage of Ad-CM. (C) IGF-1R levels in B16F1 and E0771 cells were compared by western blot, β-actin served as loading control. (D) B16F1( 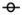) and E0771 ( 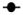) cells were cultured for 3 days in either Ad-CM or Ad-CM supplemented with different concentrations of IGF-1R inhibitor OSI-906. Cell numbers were quantified on day 3 and averaged from three independent experiments. Data was presented as percentage of Ad-CM. ^*^p<0.05. n.s., not significant.

HSC-Ad secreted a wide range of cytokines (Figure 1D, Supplementary Figure S1). To determine which factor(s) mediates B16F1 and/or E0771 cell proliferation, we evaluated several cytokines that were present in Ad-CM, including HGF, IGF-1 and VEGF. Neither of the recombinant proteins for HGF or VEGF promoted tumor cell proliferation, nor did their neutralizing antibodies suppress Ad-CM-promoted tumor cell proliferation (Supplementary Figure S2). However, recombinant IGF-1 (rIGF-1) at 100 ng/mL stimulated E0771 cell proliferation compared with αMEM alone (p<0.05) (Figure 5A). Addition of IGF-1 neutralizing antibody (1 μg/mL) suppressed Ad-CM-induced E0771 cell proliferation at day 3 by ~50% (p<0.05) (Figure 5B). In contrast, rIGF-1 did not stimulate B16F1 cell proliferation (Figure 5A), nor did IGF-1 neutralizing antibody suppress Ad-CM-induced B16F1 cell proliferation (Figure 5B). IGF-1 binds at high affinity to the IGF-1 receptor (IGF-1R) that mediates the effects of IGF-1. Western blot confirmed that E0771 cells expressed significantly higher levels of IGF-1Rβ than B16F1 cells (Figure 5C). Accordingly, a selective IGF-1R inhibitor OSI-906 (Linsitinib) suppressed Ad-CM-induced E0771 cell proliferation in a dose-dependent manner (IC50 ~30 nM), but had minimal impact on B16F1 cell proliferation (Figure 5D). These results indicate that the IGF-1/IGF-1R signaling axis is important for Ad-CM-induced E0771 cell proliferation. Unfortunately, a singular factor secreted by HSC-Ad that induced a profound stimulatory effect on B16F1 proliferation could not be identified in the studies herein.

### HSC-Adipocytes secrete HGF that accelerates cancer cell migration

**Figure 6:**
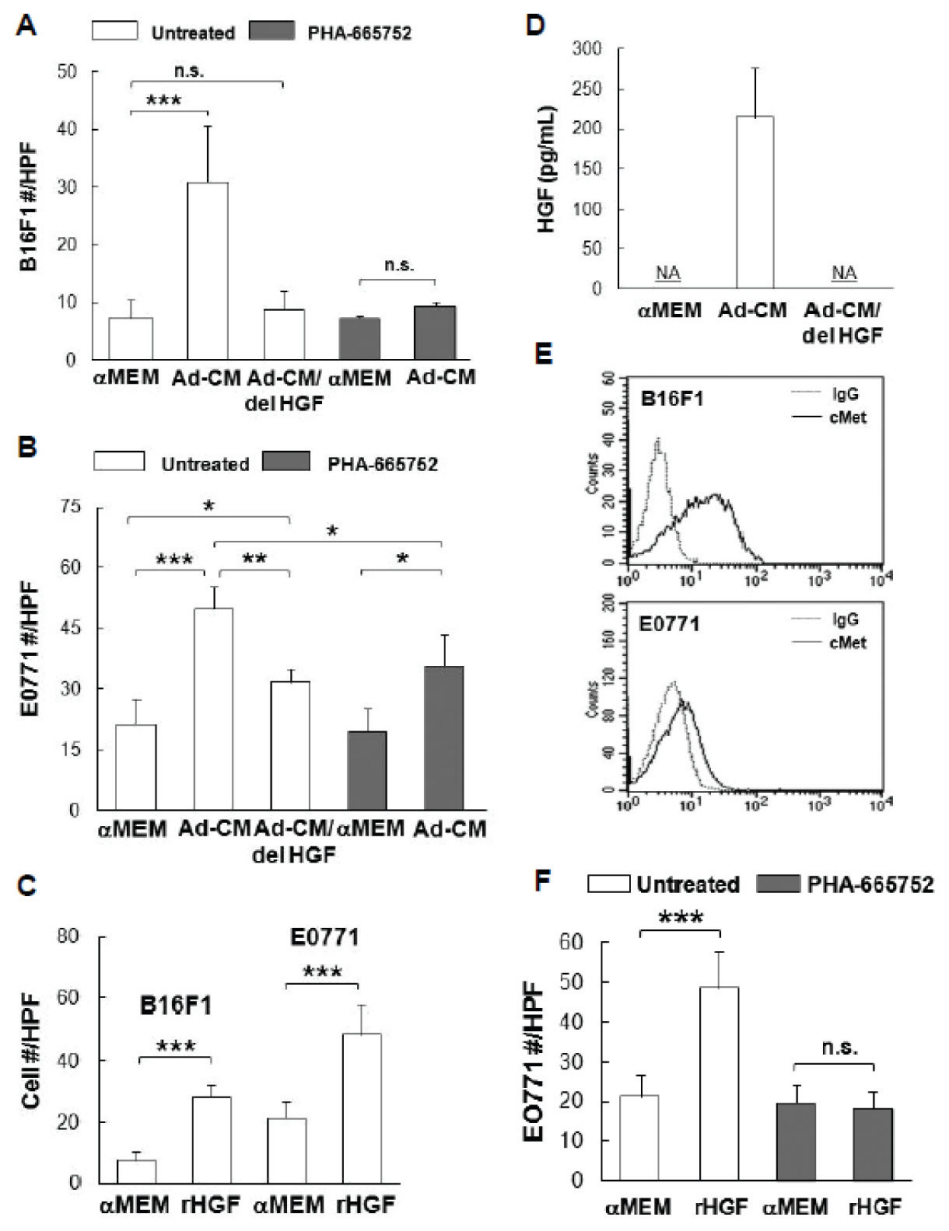
Hepatocyte growth factor (HGF)/c-Met is important for B16F1 and E0771 cell migration**-**(A) B16F1 cells and (B) E0771 cells, untreated or treated with 1 μM c-Met inhibitor PHA-665752, were exposed to the indicated medium for 4 h. Migrated cells were counted and cell # were averaged from 10 HPFs from multiple experiments. Ad-CM/del HGF, immune-depletion of HGF from Ad-CM. (C) Tumor cells were exposed to 100 ng/mL of recombinant HGF (rHGF) for 4 h. Migrated cells were counted and averaged from multiple experiments. (D) Concentrations of HGF in the indicated medium were quantified and averaged from multiple experiments with HGF ELISA kit (R&D Systems). NA, not detected. (E) Surface c-Met levels (solid lines) of tumor cells were compared by FACS analysis, isotype IgG (dash lines) served as control. (F) E0771 cells, untreated or treated with 1 μM PHA-665752, were exposed to 100 ng/mL of rHGF for 4 h. Migrated cells were counted and averaged from multiple experiments. ^*^p≤0.05, ^**^p<0.01, ^***^p<0.001. n.s., not significant.

In addition to tumor growth, tumor cell migration is an important aspect of tumor metastasis and progression, thus the effects of HSC-derived adipocytes on tumor cell motility were also examined *in vitro*. As shown in Figure 6, Ad-CM promoted the migration of B16F1 cells (Figure 6A) and E0771 cells (Figure 6B) to a higher degree than control αMEM alone (p<0.001, respectively). A number of factors secreted by HSC-Ad were tested for their ability to mediate B16F1 and E0771 cell migration. We observed that recombinant HGF (rHGF) effectively promoted both B16F1 and E0771 cell migration compared with αMEM alone (p<0.001) (Figure 6C) and HGF was present in Ad-CM (Figure 6D). Depletion of HGF from Ad-CM with anti-HGF antibody abolished Ad-CM-induced B16F1 cell migration (Figure 6A) and also significantly suppressed Ad-CM-induced E0771 cell migration (p<0.01) (Figure 6B), however, the suppression was partial compared with αMEM alone (p<0.05) (Figure 6B). The complete depletion of secreted HGF from Ad-CM was confirmed by ELISA (Figure 6D).

Receptor tyrosine kinase (RTK) c-Met is the surface receptor for HGF and mediates the effects of HGF. Using flow cytometry, we found both B16F1 and E0771 cells expressed c-Met on the cell surface, with B16F1 cells expressing significantly more c-Met than E0771 cells (Figure 6E). When B16F1 and E0771 cells were treated with c-Met selective inhibitor PHA-665752 (1 μM), Ad-CM-induced B16F1 cell migration was totally abolished (Figure 6A), whereas E0771 cell migration was only partially suppressed compared to Ad-CM alone (p<0.05) (Figure 6B). E0771 cell migration toward rHGF was abolished upon PHA-665752 treatment (Figure 6F), which confirmed the complete blockage of HGF/c-Met pathway and suggested that Ad-CM contains factor(s) other than HGF that contribute to E0771 migration. Nevertheless, these results revealed the critical role of HGF/c-Met signaling axis in B16F1 and E0771 cell motility as directed by HSC-Ad.

### HSC-Adipocytes secrete platelet-derived growth factor (PDGF) that mediates breast cancer cell migration

To reveal other factor(s) that mediate E0771 cell migration, E0771 cells were stimulated with Ad-CM and lysates were subject to a phospho-RTK array (R&D Systems), which indicated that PDGF α- and β-tyrosine kinase receptors (PDGFR and PDGFRβ) were activated and tyrosine-phosphorylated (Supplementary Figure S3A-left panel). In contrast, increased phospho-c-Met was the major event detected in Ad-CM-stimulated B16F1 cells, strengthening the significance of HGF/c-Met pathway (Supplementary Figure S3A-right panel). To validate the array data, E0771 cells were stimulated with Ad-CM for varying time points and lysates were blotted with antibodies to phospho-PDGFRβ and PDGFRβ. Western blot confirmed that Ad-CM stimulation indeed resulted in PDGFRβ tyrosine-phosphorylation and receptor degradation, two hallmarks of RTK activation (Supplementary Figure S3B).

**Figure 7:**
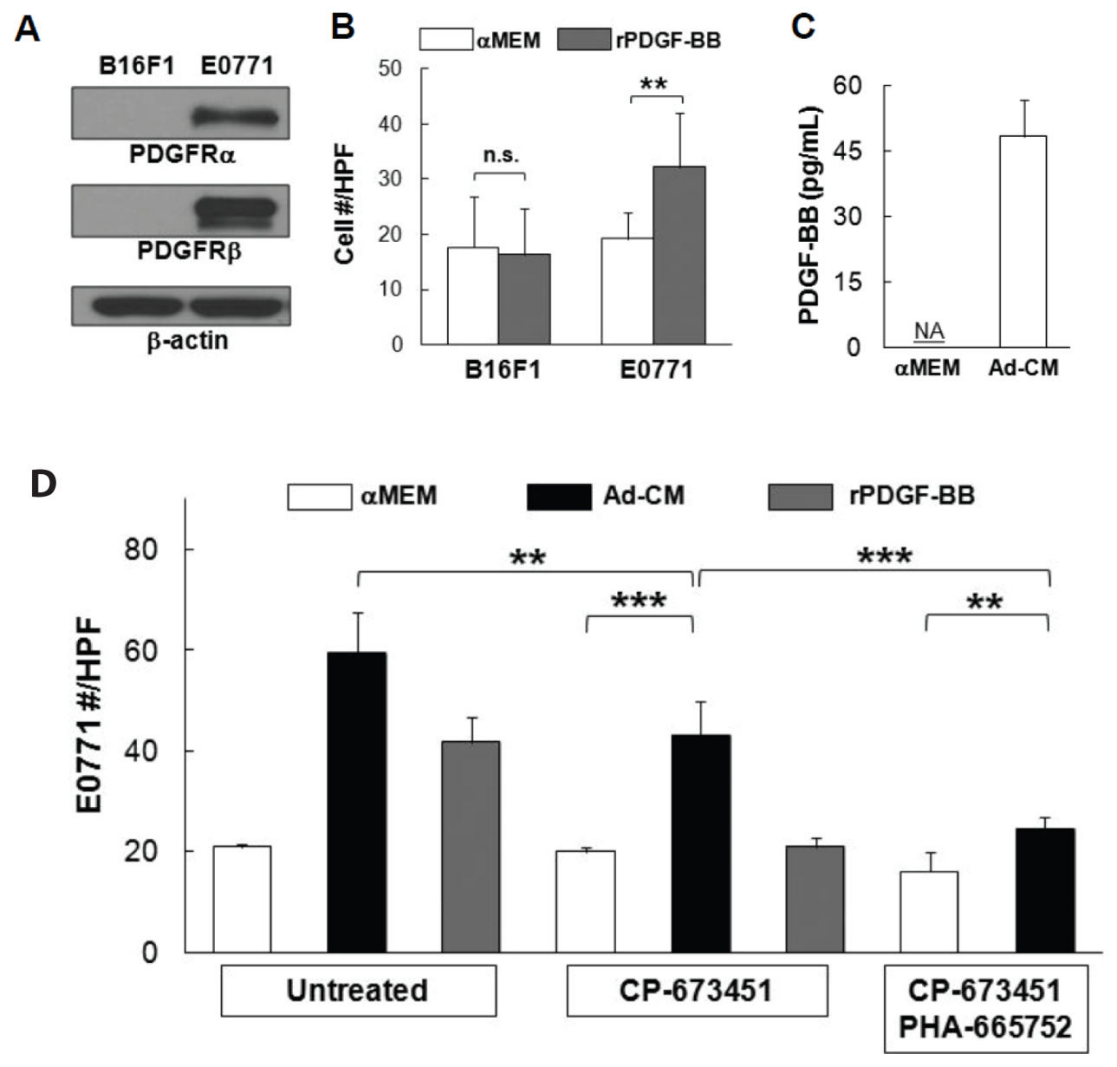
Platelet-derived growth factor-BB (PDGF-BB) mediates E0771 cell migration**-**(A) PDGF receptor α/β (PDGFRα/β) levels in B16F1 and E0771 cells were compared with western blot, β-actin served as loading control. (B) Tumor cells were exposed to αMEM or 100 ng/mL of recombinant PDGF-BB (rPDGF-BB) for 4 h. Migrated cells were counted and averaged from multiple experiments. (D) Concentrations of PDGF-BB in three independent Ad-CM preparations were quantified and averaged with PDGF-BB ELISA kit (R&D Systems). NA, not detected. (D) E0771 cells, untreated or treated with 100 nM PDGFRα/β inhibitor CP-673451 or a combination of 1 μM PHA-665752 and 100 nM CP-673451, were exposed to the indicated medium for 4 hr. Migrated cells were counted and averaged from multiple experiments. ^**^p<0.01, ^***^p<0.001. n.s., not significant.

The PDGF family consists of disulfide-bonded homodimers of A-, B-, C- and D-polypeptide chains and the heterodimer PDGF-AB. PDGF isoforms exert their biological functions through binding and activation of PDGFRα and PDGFRβ, which are involved in the progression of a number of tumors, including breast cancer [26]. Western blot demonstrated the expression of PDGFRα and PDGFRβ in E0771 cells, which is undetectable in B16F1 cells (Figure 7A). Recombinant PDGF-BB (rPDGF-BB) was able to promote E0771, but not B16F1 cell migration compared with αMEM alone (p<0.01) (Figure 7B). PDGF-BB was detected in Ad-CM (Figure 7C).

When E0771 cells were treated with PDGFRα/β selective inhibitor CP-673451 (100 nM), Ad-CM-induced E0771 cell migration was significantly compromised (p<0.01), but the suppression was partial (p<0.001) (Figure 7D). Meanwhile, CP-673451 treatment completely abolished E0771 cell migration toward rPDGF-BB, demonstrating the complete blockage of PDGFR activation (Figure 7D). When E0771 cells were treated with a combination of CP-673451 and PHA-665752, Ad-CM-induced E0771 cell migration was further suppressed (p<0.001) (Figure 7D). It is noted that CP-673451 and PHA-665752 together did not completely abolish Ad-CM-induced E0771 migration (p<0.01) (Figure 7D), suggesting that factor(s) other than HGF and PDGF-BB may also play a role in E0771 cell migration *in vitro*.

## Discussion

Herein, we have studied the differentiation and maturation of HSC-derived adipocytes from monocytic precursors and their potential contributions to cancer progression. The generation of monocytic precursors is dependent on M-CSF [27]. In mice deficient in M-CSF or M-CSF receptor, the number of blood monocytes and tissue macrophages is dramatically reduced [28,29]. Interestingly, M-CSF also participates in adipocyte hyperplasia/hypertrophy and the physiological regulation of adipose tissue growth [30,31], highlighting the involvement of monocytic cells in adipose tissue development. Our results demonstrate that monocytic precursors generate immature adipocytes with multilocular oil droplets, which are able to undergo further maturation when subjected to an appropriate adipose environment. Mature HSC-Ad does not possess the expression of hematopoietic marker CD45 [19].

Tumor vascularization is believed to reinforce oxygen and nutrient supply to the tumor and facilitate tumor growth. This process is regulated by a variety of pro-angiogenic factors, including VEGF, a general activator of endothelial cell and progenitor cell proliferation [32]. Our observation that co-injection of HSC-Ad with tumor cells resulting in enlarged blood vessel lumen is likely attributed to HSC-Ad-secreted VEGF, which can increase the number of the endothelial cells lining around the blood vessels, thus allowing the expansion of the lumen area.

HSC-Ad secreted growth factors and adipokines that exert tumor-type specific effects. Regarding cancer cell proliferation, tumor growth, and tumor vascularization, B16F1 melanoma cells were more responsive to HSC-Ad influence, compared with E0771 breast cancer cells. However, none of the individual factors tested in the study showed determinant contribution to B16F1 cell proliferation. We speculate that HSC-Ad mediate B16F1 tumor growth *via* collective efforts from multiple growth factors and their cognate receptors, without any one pathway being dominant. On the other hand, E0771 cells responded to IGF-1 stimulation and expressed much higher level of IGF-1R than B16F1 cells, suggesting that HSC-Ad rely more on the IGF-1/IGF-1R signaling pathway to regulate E0771 tumor growth. Selective inhibitor of the IGF-1R kinase has been shown to inhibit survival and anchorage-independent growth of MCF-7 human breast cancer cells [33]. Currently, more than ten IGF/IGF-1R inhibitors are under clinical investigation for various cancers, including breast cancer [34]. Our findings suggest another mechanism by which these inhibitors may function.

Cell migration is a critical aspect of cancer progression that is linked with metastasis. The cell surface receptor tyrosine kinases, c-Met and PDGFR, upon binding of their ligands, activate multiple signaling cascades that induce cell growth, survival, and motility [35,36]. Hyperactivity of the HGF/c-Met and PDGF/PDGFR signaling pathways occur in different cancer types and have been associated with the uncontrolled growth of tumor cells, epithelial-to-mesenchymal transition, invasiveness, and metastasis [37,38]. Small-molecule inhibitors targeting c-Met tyrosine kinase activity have been shown to prevent metastasis in several cancer models, including melanoma [39,40] and breast cancer [41,42]. We demonstrated that HSC-Ad enhanced B16F1 cell migration primarily through the HGF/c-Met signaling axis, whereas in addition to the HGF/c-Met axis, the PDGF/PDGFR pathway contributed significantly to E0771 cell migration promoted by HSC-derived adipocytes. The influence of PDGF/PDGFR signaling could be due to higher expression of PDGFRβ and lower expression of c-Met in E0771 cells compared with B16F1 cells, so that the HGF/c-Met axis is not predominant, yet still important for E0771 cell motility. It is possible that inhibition of both pathways will present better efficacy in suppressing breast cancer metastasis.

While these *in vitro* data demonstrate the potential of factors secreted from HSC-Ad to promote tumor cell proliferation and migration, it is likely that these effects are potentiated by the complex tumor microenvironment, resulting in enhanced tumor growth seen in co-injected cohorts *in vivo.*

HSC provides a novel source for adipocyte generation, yet HSC-Ad is not the major pathway for adipose tissue development under normal conditions [43]. However, whether HSC-Ad has any potential roles under pathological conditions, such as obesity and cancer, are completely unknown. We hypothesize that during the development of obesity, the elevated level of circulating chemo-attractants (e.g. MCP-1) will recruit monocytic cells to the adipose tissue and differentiate into mature adipocytes. HSC-Ad then secretes adipokines, inflammatory cytokines and chemokines that lead to further enhanced monocytic precursor recruitment, HSC-Ad accumulation, and immune cell infiltration. It has been shown that HSC-Ad preferentially accumulate in the visceral adipose tissue (VAT) over the subcutaneous adipose tissue (SAT) and in female mice more than male mice [19]. Excess adiposity in VAT is known to be particularly linked to type 2 diabetes and certain forms of cancer, whereas excess SAT confers a much lower risk for these conditions [44]. Moreover, humans generally tend to disproportionally gain fat in VAT, and increased body-mass index is positively associated with melanoma and breast cancer [45–47]. Thus, HSC-Ads are likely to play an integral role in adipose-related tumor progression, and represent a higher risk of adipose-related gynecological cancers for women. HSC-Ad may represent a novel target for therapies aimed at inhibiting the increased aggressiveness and metastatic capabilities of cancer cells especially in obese patients. More importantly, our works suggest that future therapeutic strategies for treatment of adipose-related cancers should be depot- and tumor type-dependent.

## Supplementary Material
