# Supplementary material for: Hematopoietic Stem Cell-derived Adipocytes Promote Tumor Growth and Cancer Cell Migration
Source: Int J Cancer Res Mol Mech. Author manuscript; Available in PMC 2017 Oct 4. (PMC5627654; doi:10.16966/2381-3318.130)
Supplement: Supplementary file 1 [file NIHMS859913-supplement-supplement_1.pdf]

Supplementary Information

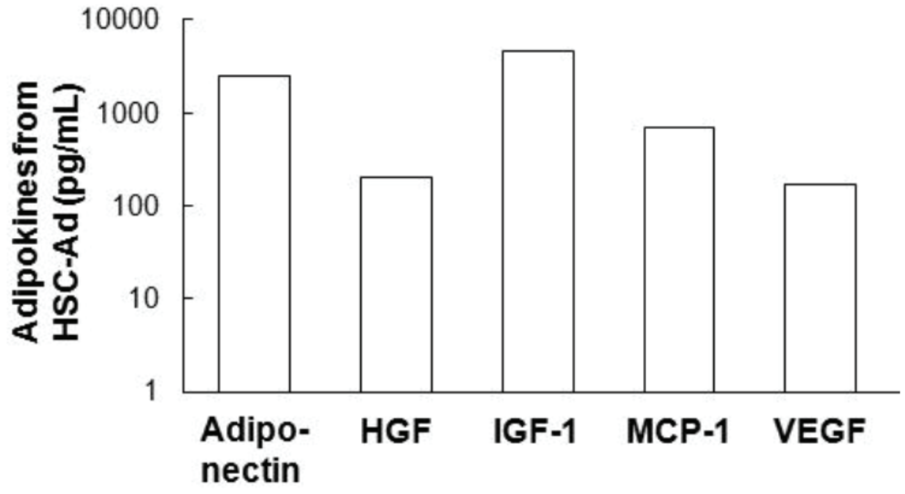

Supplementary Figure S1: HSC-Ad secretes adipokines-Ad-CM was subject to individual ELISAs (R&D Systems). The indicated adipokines were confirmed and quantified.

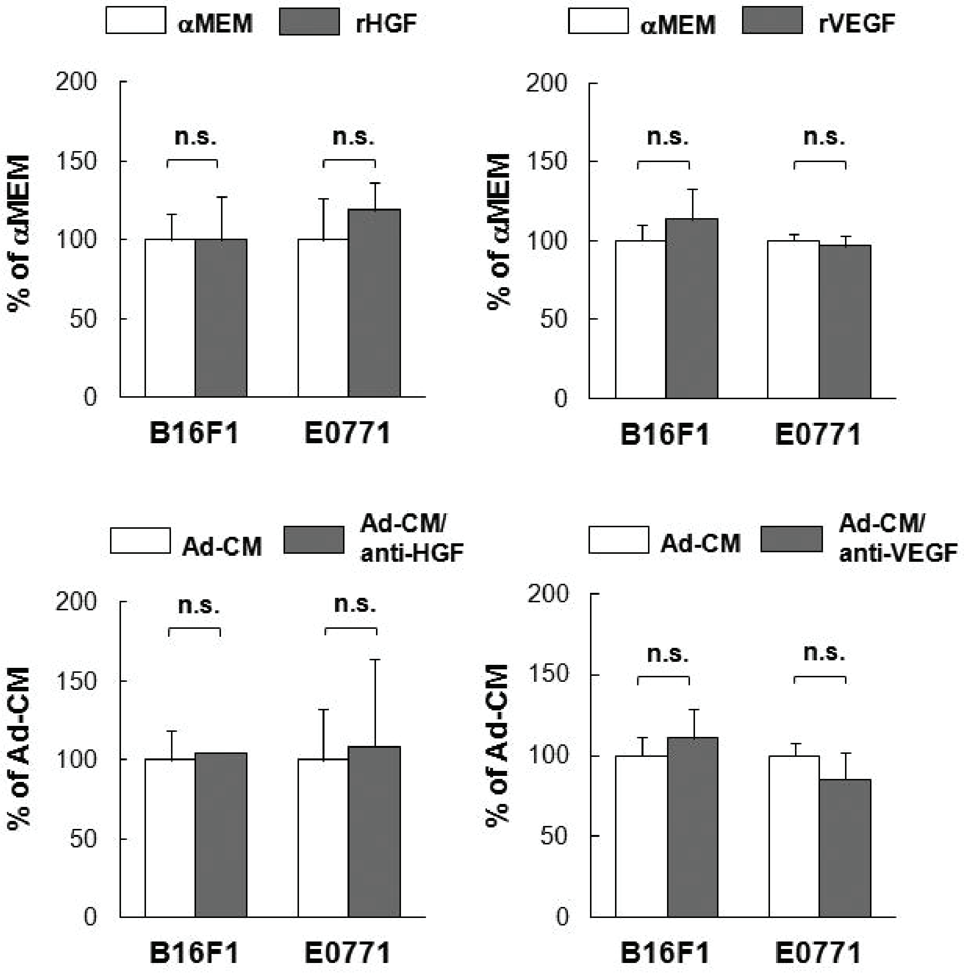

Supplementary Figure S2: HGF and VEGF are dispensable for Ad-CM-supported tumor cell proliferation-Tumor cells were cultured for 3 days in (A) αMEM or 100 ng/mL of HGF or VEGF, and (B) Ad-CM or Ad-CM supplemented with 1 µg/mL of anti-HGF or anti-VEGF neutralizing antibodies. Cell numbers were quantified and presented as percentage of αMEM (A) and Ad-CM (B). No significant differences were observed.

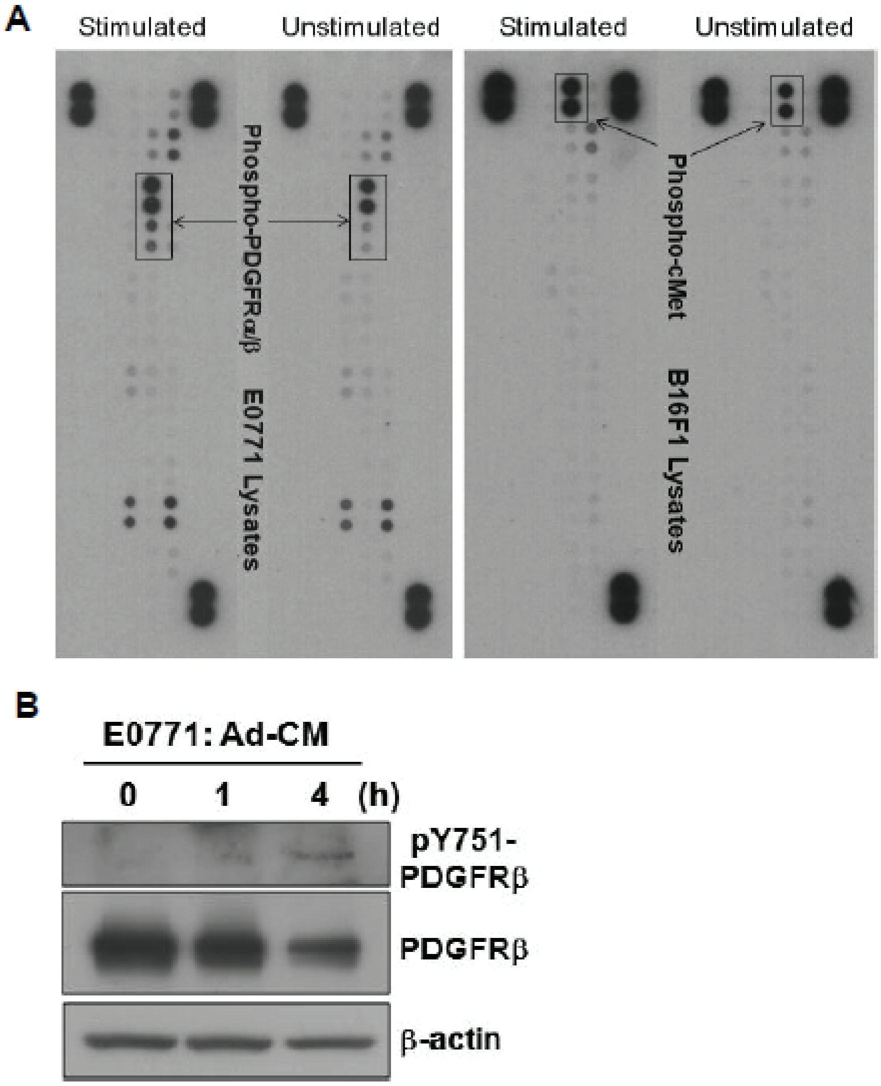

Supplementary Figure S3: Ad-CM activates PDGFR signaling-(A)Tumor cells were untreated or treated with Ad-CM at 37°C for 5 min and cell lysates were applied to a Phospho-RTK Array (R&D Systems). Data indicated that Ad-CM stimulation can increase phospho-PDGFRα/β level in E0771 cells (left panel) and increase phospho-cMet level in B16F1 cells (right panel). (B) E0771 cells were untreated or treated with Ad-CM at 37°C for the indicated times. Cell lysates were subject to SDS-PAGE and blotted with the indicated antibodies. Data confirmed Ad-CM-stimulated tyrosine phosphorylation and activation of PDGFRβ in E0771.

Citation: Xiong Y, Russell DL, McDonald LT, Cowart LA, LaRue AC (2017) Hematopoietic Stem Cell-derived Adipocytes Promote Tumor Growth and Cancer Cell Migration. Int J Cancer Res Mol Mech 3(1): doi http://dx.doi.org/10.16966/2381-3318.130
